# AttenRNA: multi-scale deep attentive model with RNA feature variability analysis

**DOI:** 10.1093/bib/bbaf336

**Published:** 2025-07-10

**Authors:** Jing Li, Quan Zou, Chao Zhan

**Affiliations:** Yangtze Delta Region Institute (Quzhou), University of Electronic Science and Technology of China, Chengdian Road, Minjiang Avenue, Kecheng District, Quzhou, Zhejiang Province, 324000, China; Department of Microbiology, University of Hong Kong, Block T, Queen Mary Hospital, Pok Fu Lam Road, Pok Fu Lam, Hong Kong SAR, China; School of Biomedical Sciences, University of Hong Kong, Laboratory Block, 21 Sassoon Road, Pok Fu Lam, Hong Kong SAR, China; Yangtze Delta Region Institute (Quzhou), University of Electronic Science and Technology of China, Chengdian Road, Minjiang Avenue, Kecheng District, Quzhou, Zhejiang Province, 324000, China; Institute of Fundamental and Frontier Sciences, University of Electronic Science and Technology of China, 2006 Xiyuan Avenue, Western Hi-Tech Zone, Chengdu, Sichuan Province, 611731, China; Department of Hepatopancreatobiliary Surgery, Harbin Medical University Cancer Hospital, Nangang District, Harbin, Heilongjiang, 150086, China

**Keywords:** circRNA, lncRNA, mRNA, attention mechanism, multi-scale k-mer modules, multi-class classification

## Abstract

Accurate identification of diverse RNA types, including messenger RNAs (mRNAs), long non-coding RNAs (lncRNAs), and circular RNAs (circRNAs), is essential for understanding their roles in gene regulation, disease progression, and epigenetic modification. Existing studies have primarily focused on binary classification tasks, such as distinguishing lncRNAs from mRNAs or identifying specific circRNAs, often overlooking the complex sequence patterns shared across multiple RNA types. To address this limitation, we developed AttenRNA, a multi-class classification model that integrates multi-scale k-mer embeddings and attention mechanisms to simultaneously differentiate between various RNA classes. AttenRNA achieved high weighted F1 scores of 89.8% and 89.6% on the validation and test sets, respectively, demonstrating strong classification performance and robustness. Dimensionality reduction using Uniform Manifold Approximation and Projection further confirmed the model’s ability to learn discriminative features among RNA types. Additionally, AttenRNA exhibited strong generalization ability on cross-species data, achieving weighted F1 scores of 83.89% and 83.38% on the mouse RNA validation and test sets, respectively. These results suggest that AttenRNA offers a reliable and scalable solution for systematic RNA function analysis.

## Introduction

RNA in eukaryotes is divided into coding RNA and non-coding RNA (ncRNA), with non-coding RNA comprising short non-coding RNA and long non-coding RNA (lncRNA) [1, 2]. LncRNAs are transcripts with a length of >200 nucleotides [3]. In recent years, lncRNAs have received extensive attention owing to their key roles in gene expression regulation, epigenetic modification, chromatin remodelling, and other biological processes [4, 5]. In addition, lncRNAs play an important role in the occurrence and progression of multiple diseases, such as cancer and cardiovascular diseases, and are potential therapeutic targets and diagnostic markers [6–8]. Circular RNAs (circRNAs) have high stability owing to their closed-ring structure, which enables them to avoid exonuclease degradation [9, 10]. CircRNAs not only indirectly regulate mRNA expression through the miRNA sponge mechanism, but also participate in the occurrence and development of many diseases, including cancer and neurodegenerative diseases [11, 12]. In recent years, with the development of RNA sequencing (RNA-seq) technology, many circRNAs and lncRNAs have been identified in various species [13–15]. However, because of their low expression levels and overlapping features with other RNA types, precise distinction of RNA types remains challenging [16, 17]. Messenger RNA (mRNA), the coding RNA responsible for carrying genetic information to guide protein synthesis, is considerably different from circRNAs and lncRNA [18, 19]. However, from the sequence point of view, mRNA shares many similar features with lncRNA, such as a 5′ end-cap structure, a 3′ end poly(A) tail, and variable splicing; therefore, it is complicated to distinguish mRNA from lncRNA at the sequence level. Efficient classification models not only help to understand the functional differences between different RNA types, but also provide support for further research on the relationship between RNA and diseases [20–24].

Recently, RNA molecules, particularly non-coding RNAs, have emerged as a crucial area of research in molecular biology. CircDC [25] uses a convolutional neural network and bidirectional LSTM to distinguish circRNAs from other lncRNAs, demonstrating higher accuracy than existing models. WebCircRNA [26] describes a user-friendly web server that uses random forest models to predict whether coding and non-coding RNAs have circRNA isoforms and to assess their expression in stem cells. Zhang *et al*. [27] proposed a novel method using a Class Similarity Network to explore the potential relationships among input samples in coding RNA and lncRNA classification. LncDC [28] accurately predicts lncRNAs using an XGBoost model that incorporates RNA sequences, secondary structures, and protein translation features. LncRNAnet [29] combines recurrent neural networks and convolutional neural networks to identify lncRNAs and mRNAs. DeepLNC [30] describes a deep neural network classifier using k-mer information content to accurately identify lncRNAs and mRNAs using an associated web prediction tool available online. PLEK [31] is an alignment-free tool based on an improved k-mer scheme and an support vector machine (SVM) algorithm [32, 33] that distinguishes lncRNAs from mRNAs, and is suitable for species without reference genomes. These studies have generally utilized machine learning and deep learning approaches to differentiate various RNA types, such as lncRNAs, mRNA, circRNAs, and lncRNAs [34, 35]. Studies based on the methods listed above demonstrated the potential applications of sequence features and advanced algorithmic models for RNA classification. However, they mostly focus on binary classification tasks, such as distinguishing between lncRNAs and mRNA or identifying specific types of circRNAs, often failing to capture more complex patterns within sequences. In fact, circRNAs, lncRNAs, and mRNAs play distinct roles in cellular functions and regulatory mechanisms. A multi-class classification framework enables a more comprehensive understanding of their functional differences and interrelationships, potentially uncovering their diverse roles in disease progression and biological processes. From a clinical perspective, accurately distinguishing among these RNA types can provide more precise guidance for disease diagnosis and therapeutic decision-making. Currently, no model is capable of simultaneously classifying circRNAs, lncRNAs, or mRNA. This is an urgent task that needs to be addressed and its resolution will provide deeper insights into the complex roles of RNA in various biological processes. The development of new models and frameworks capable of handling complex classification tasks is a key direction for future research.

To solve this problem, we propose AttenRNA, a deep learning classification model for the simultaneous classification of circRNAs, lncRNAs, and mRNA. AttenRNA can comprehensively capture short- and medium-range nucleotide patterns in RNA sequences through a multi-scale k-mer embedding method and attention mechanism, and can dynamically evaluate the importance of different features, thus achieving accurate classification of the three RNA types. Compared with existing methods, AttenRNA can not only handle complex multi-classification tasks, but also considerably improves classification performance. The experimental results show that the F1-score-weighted AttenRNA in the validation and test sets reached 89.8% and 89.6%, respectively, which is significantly better than that of other baseline models. AttenRNAs not only delivers advances in RNA classification but also provides valuable insights for understanding the characteristic distinctions of different RNA types, which lays a solid foundation for subsequent RNA function studies and the identification of disease-related RNAs.

## Methods

### Data description

**Figure 1:**
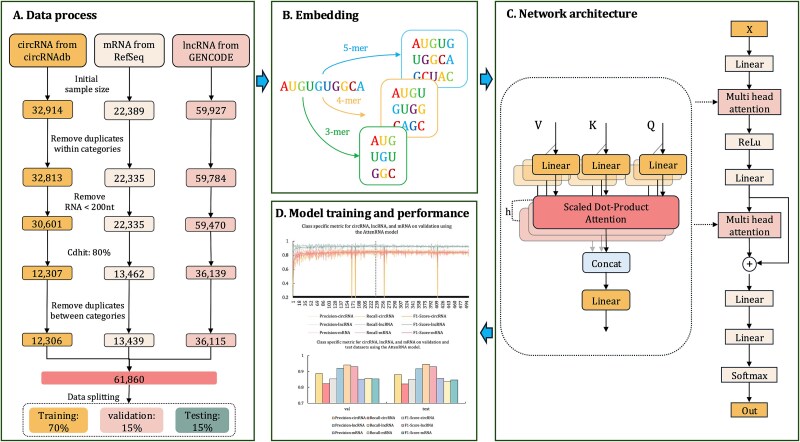
The flowchart of AttenRNA. (A) Data process. (B) Multi-scale k-mer embedding. (C) Network architecture: The AttenRNA model employs a multi-head attention mechanism. (D) Model training and performance. Metrics on the validation and test datasets for circRNA, mRNA, and lncRNA classification are presented. The upper panel shows the metric trends over epochs for validation data, while the lower panel displays precision, recall, and F1 scores for each RNA type on validation and test datasets. These results demonstrate the effectiveness of AttenRNA in classifying RNA sequences.

For the circRNA data, we analysed circRNA sequences from the CircBase (https://www.circbase.org/) [36] and CircRNADb (http://reprod.njmu.edu.cn/cgi-bin/circrnadb/circRNADb.php) [37] databases. The results showed that the circRNADb database contains all the circRNA sequences in the circBase database, indicating that the circRNADb database has more comprehensive coverage. In a follow-up study, we analysed only 32 914 circRNA sequences from the circRNADb. We removed the repeats of circRNADb to obtain 32 813 circRNA sequences. Considering that a circRNA channel that is too short cannot effectively form a stable ring node, we removed circRNA sequences <200 nt among them and 30 601 circRNA sequences. Next, Cdhit was used to remove redundant data and the similarity threshold was set to 80%. Finally, 12 307 circRNA sequences were obtained. We performed the above procedures on 22 389 mRNA sequences from RefSeq [38, 39], including removing repeated RNA sequences, removing sequences with RNA <200 nt, and setting the threshold of cdhit to 80% to obtain 13 462 mRNA sequence samples. We performed the same procedure for 59 927 lncRNAs from GENCODE (https://www.gencodegenes.org/) [40, 41], resulting in 36 139 lncRNA sequences (Fig. 1A). To ensure the independence and accuracy of the data, we conducted intergroup deduplication for the three types of RNA datasets, and we obtained 12 306 circRNA, 13 439 mRNA, and 36 115 lncRNA sequences, which laid a reliable foundation for subsequent analyses. To further evaluate the generalizability of our method across species and verify its robustness in different biological contexts, we additionally incorporated mouse RNA data into the analysis. We obtained mouse circRNA, lncRNA, and mRNA samples from the same database and applied the same data processing methods. As a result, the number of mouse circRNA, lncRNA, and mRNA samples was 10 664, 19 020, and 5465, respectively. The human and mouse dataset were then divided into training, validation, and test sets, with 70% used for training, 15% for validation, and 15% for testing. The distribution of human and mouse circRNAs, lncRNAs, and mRNAs in the training set, validation set, and test set is shown in Table 1. In this study, we used the F1-score-weighted with comprehensive performance as the primary evaluation indicator. The F1-score-weighted effectively balances the impact of the imbalance among different class samples, making the evaluation results more reflective of the model’s overall performance across all categories.

**Table 1:** Data distribution of human and mouse RNA types across training, validation, and test sets

		Training	Validation	Test
Human	circRNA	8614	1846	1846
	lncRNA	25 282	5417	5417
	mRNA	9407	2016	2016
Mouse	circRNA	7465	1599	1600
	lncRNA	13 314	2853	2853
	mRNA	3825	820	820

### Description of the proposed AttenRNA

To construct a model that effectively identifies the differences between various types of RNA, we employed a multi-scale k-mer (Fig. 1B). Specifically, by extracting features from 3-mer, 4-mer, and 5-mer RNA sequences, we captured both short- and medium-long-range nucleotide sequence patterns to reflect the sequence characteristics of different RNA categories. Through this multi-scale feature extraction, we hope to comprehensively capture sequence differences among circRNAs, mRNA, and lncRNAs and provide rich feature representations for subsequent classification tasks. The network framework is shown in Fig. 1C. The core uses a multi-head attention mechanism that focuses on key patterns in the sequence by extracting correlations between embedded features. Specifically, the input-embedded features were linearly transformed to generate queries (Q), keys (K), and values (V), and a Scaled Dot-Product Attention module was used to calculate the weights between locations, thereby capturing higher-order dependencies between features. Then, through feature fusion and multilayer linear transformation, the model further extracts sequence information and performs classification prediction. This network structure effectively utilized embedded features to capture the differences between RNA sequences, providing strong support for the accurate identification of different RNA types.

For AttenRNA, we performed a statistical analysis of the changes in the weights F1, macro F1, and Accuracy of the validation set to evaluate the performance and convergence of the model over different periods (see Fig. 1D). With an increase in epochs, the F1 weight, F1 macro, and accuracy show an upward trend and gradually become stable. This trend is consistent with the gradual optimization of the model training. The model achieved the highest F1-score-weighted value when the number of epochs was 235. In addition, Fig. 1D shows the fluctuations in the AttenRNA indicators in the validation set for the three types of RNAs. In the early stages of training, various indicators fluctuated greatly, especially circRNAs. However, as the training iterations progressed, all metrics gradually stabilized, ultimately achieving high performance levels in the validation set. For mRNA and lncRNA, the accuracy and F1 of the model remained high, and the recall was relatively stable, indicating that the model could stably and accurately identify these two types of RNA. For circRNAs, although the initial fluctuation was large, their indicators also reached a level close to that of the mRNA after convergence. Overall, the model showed a high classification ability for all three RNA types, verifying the generalization of AttenRNA and its ability to capture the features of different RNA classes.

### Multi-scale k-mer modules

k-Mer embedding is a method for converting a biological sequence (e.g. DNA, RNA, or protein) into numerical features, capturing the local information of the sequence by extracting a continuous subsequence of a fixed length (called a k-mer). The core idea is to numerically represent the content and arrangement of sequences, which facilitates the input and processing of machine learning or deep learning models. To characterize the diversity and complexity of RNA sequence features more comprehensively, we designed a multi-scale k-mer module. Specifically, the module extracts the 3-mer, 4-mer, and 5-mer features of RNA sequences and combines these features to generate input data that are richer and have multi-scale feature expression. This method can simultaneously capture short-distance (such as local sequence patterns represented by 3-mer) and medium-distance (such as global sequence structures represented by 4-mer and 5-mer) information, thereby enhancing the ability of the model to recognize RNA sequence differences at the feature level. Multi-scale k-mer feature fusion not only improves the diversity of model input data but also provides a more expressive feature representation for subsequent classification tasks.

### Calculation and statistical analysis of GC content

To explore the differences in the sequence composition of circRNAs, lncRNAs, and mRNA, GC content was calculated and statistically analysed. GC content was calculated using the following formula:


(1) \begin{equation*} CG\ content=\frac{number\ of\ C+ number\ of\ G}{total\ sequence\ length} \end{equation*}

This formula was used to measure the proportion of C and G bases in the RNA sequence, which is an important indicator of the compositional characteristics of the RNA sequence and may be related to its function and stability. The H-statistic of the Kruskal–Wallis test was 1575.1943, and the *P*-value was <.0001, indicating the significance of the difference between RNA groups. These results collectively validated the significant differences in sequence composition among the different RNA types.

### UMAP visualization

In order to explore the feature distribution of circRNA, mRNA, and lncRNA, we used Uniform Manifold Approximation and Projection (UMAP) [42, 43] to perform reduction and visualization analysis of multi-scale k-mer. UMAP is a nonlinear dimensionality reduction technique that preserves both local and global structural information in high-dimensional feature spaces, thereby helping us to observe the distribution characteristics of data in low-dimensional space more intuitively. To eliminate the difference of feature dimension, we standardize the multi-scale k-mer features so that they have zero mean and unit standard deviation. Subsequently, UMAP is used to reduce the high-dimensional features to two dimensions, with a neighbourhood size of 80 a minimum distance of 0.005, and the metric used is Chebyshev distance. The results of dimension reduction are shown in scatter plots. Different colours mark RNA categories: green represents circRNA, red represents lncRNA, and blue represents mRNA. The visualization results intuitively show the clustering distribution of the three RNA types in two-dimensional space, providing important evidence for study of differences in RNA.

### Evaluation metrics

In this study, in order to comprehensively evaluate the performance of the model in different RNA type classification tasks, we used a variety of evaluation indicators, including the overall performance indicators of the multi-classification tasks and individual indicators for each RNA type. In the overall metric of multi-classification tasks, precision (macro/weighted) measures the model’s accuracy in predicting each class, precision-macro calculates the simple average of precision across all classes, treating all classes equally (Formula 2), whereas precision-weighted accounts for the number of samples in each class, providing a size-weighted average (Formula 3). Recall (macro/weighted) indicates the ability of the model to correctly identify all actual positives for each class. Similar to precision, the recall macro averages the recall across all classes (Formula 4), and the recall-weighted macro averages the recall based on the number of true instances for each class (Formula 5). The F1 Score (macro/weighted) is the harmonic mean of Precision and Recall. The F1 score balances the tradeoffs between precision and recall and serves as a crucial composite indicator of model performance in multi-class tasks. The F1-macro computes this mean across all classes equally (Formula 6), whereas the F1-weighted Score computes this mean based on the prevalence of each class (Formula 7). Accuracy is the proportion of the total correct predictions made by the model across all classes and represents the most straightforward and intuitive performance metric (Formula 8).

To further analyse the classification effect of the model on the three types of RNAs, circRNA, lncRNA, and mRNA, we calculated each RNA indicator: ‘precision’ evaluates the accuracy of the model’s predictions for a specific category, ‘recall’ assesses the model’s ability to identify all actual positive instances of a class, and ‘F1 Score’ represents the harmonic mean of precision and recall, indicating its usefulness in providing a balanced measure of the classification performance of a model for specific RNA categories. All these metrics were calculated separately for the validation and test sets to ensure the objectivity and robustness of the model performance. In addition, to address the category imbalance, we paid special attention to the weighted F1 Score as an important reference for measuring the overall performance of the model.


(2) \begin{equation*} {precision}_{macro}=\frac{1}{N}\sum_{i=1}^N\frac{TP_i}{TP_i+{FP}_i} \end{equation*}

(3) \begin{equation*} {precision}_{weighted}=\frac{1}{Total\ Samples}\sum_{i=1}^N\left({TP}_i+{FP}_i\right)\ast \frac{TP_i}{TP_i+{FP}_i} \end{equation*}

(4) \begin{equation*} {recall}_{macro}=\frac{1}{N}\sum_{i=1}^N\frac{TP_i}{TP_i+{FN}_i} \end{equation*}

(5) \begin{equation*} {recall}_{weighted}=\frac{1}{Total\ Samples}\sum_{i=1}^N\left({TP}_i+{FN}_i\right)\ast \frac{TP_i}{TP_i+{FN}_i} \end{equation*}

(6) \begin{equation*} F{1}_{macro}=\frac{1}{N}\sum_{i=1}^N\frac{2\ast{Precision}_i\ast{Recall}_i}{Precision_i+{Recall}_i} \end{equation*}

(7) \begin{equation*} F{1}_{weighted}=\frac{1}{Total\ Samples}\sum_{i=1}^N\left({TP}_i+{FN}_i\right)\ast \frac{2\ast{Precision}_i\ast{Recall}_i}{Precision_i+{Recall}_i} \end{equation*}

(8) \begin{equation*} Accuracy=\frac{\sum_{i=1}^N{TP}_i}{Total\ Samples} \end{equation*}

where ${TP}_i$, ${FP}_i$, and ${FN}_i$ represent the numbers of true positives, false positives, and false negatives for class *i*, respectively, and *N* is the total number of classes.

## Results

### Analysis of GC content in different RNA types

**Figure 2:**
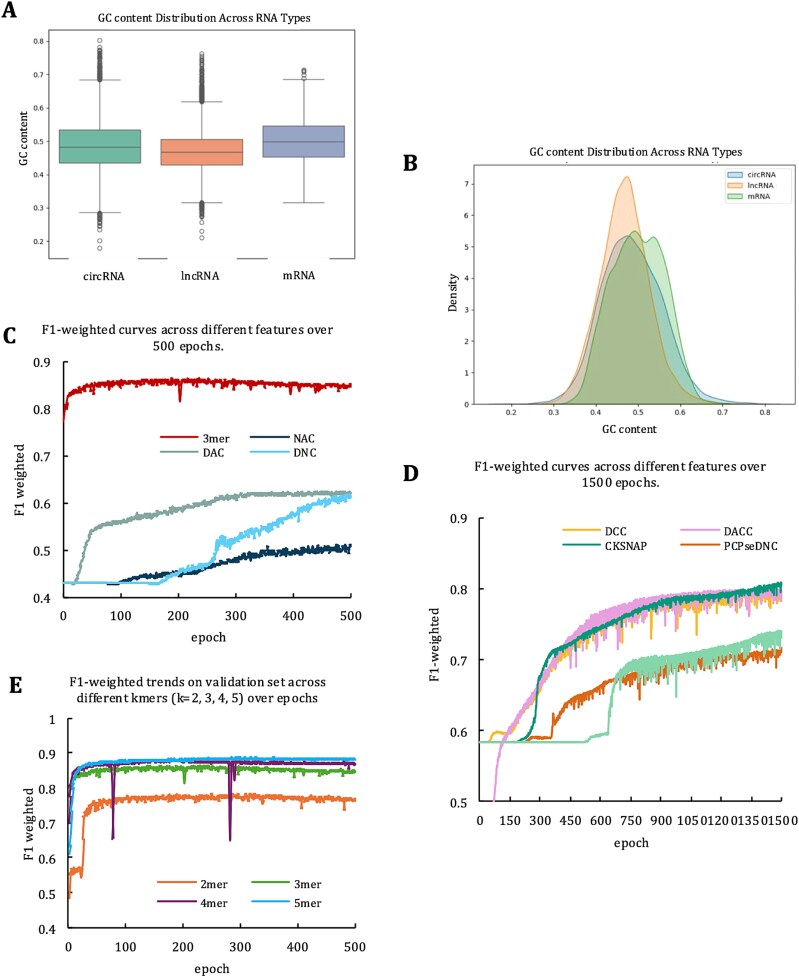
Analysis of RNA features and model performance trends. (A) Boxplot of GC content across circRNA, lncRNA, and mRNA. (B) Density plot of GC content distribution for circRNA, lncRNA, and mRNA. (C) F1-Weighted performance curves for different features >500 epochs. (D) F1-Weighted performance curves for different features >1500 epochs. (E) Validation set F1-weighted trends for various k-mer lengths (*k* = 2, 3, 4, 5) over epochs.

To explore the sequence characteristics of different RNA types, we calculated the GC content [44, 45] of three RNA types (i.e. circRNA, mRNA, and lncRNA) in the human data. GC content visualization via boxplots and density maps (Fig. 2A and 2B) revealed differences in CG distribution across the three RNA types. Figure 2A shows that there were differences in the median GC content of different RNA types, among which mRNA had the highest median GC content. The mRNAs exhibited a wider distribution range and fewer extreme values. The GC content of both human circRNAs and lncRNAs showed more extreme values. The median value of lncRNAs was the lowest among the three RNAs. As shown in Fig. 2B, both density curve and peak position (~+50%) were high for human mRNA. The distribution of the region with a high GC content (>60%) was also significant, indicating that the GC content of the mRNA was highly variable and high overall. In human circRNAs, the peak position of the density curve was between that of lncRNAs and mRNA (~48%), indicating that the GC content in circRNAs was moderate and evenly distributed. In human lncRNAs, the density curve was the narrowest and the peak position was close to the low GC content (~45%), indicating that the GC content distribution of lncRNAs was concentrated and the variability was small.

To further verify whether the difference in GC content between the different RNA types was statistically significant, we conducted a non-parametric statistical analysis (Kruskal–Wallis test). The circRNA density map in Fig. 2B shows a bimodal feature, indicating that the data may not conform to a normal distribution. The Kruskal–Wallis test [46, 47] does not require data to satisfy the premise of a normal distribution; therefore, it is well suited for working on datasets with skewed distributions or containing outliers. This makes the Kruskal–Wallis test particularly suitable for the current analysis. The Kruskal–Wallis test results showed that the difference between the three RNA types was significant (*H*-statistic = 1575.1943, *P*-value = .0000e+00). *H*-Statistic values indicated significant differences in the distribution of different RNA types.

### Feature analysis

Features were extracted at different levels. As shown in Fig. 2C, the initial F1-weighted value of the 3-mer [48, 49] single-feature model on the verification set was 77%, which then gradually increased and stabilized at 85% for the top 50 epochs. This shows that the model gradually optimizes the balance between precision and recall rate when processing datasets, thereby improving the overall classification performance. The F1-weighted value of the Nucleotide Acid Composition (NAC) [49] model was low and finally stable at ~50%, indicating that NAC features have a limited ability to differentiate. In the top 300 epochs of dinucleotide-based autocovariance (DAC) [49, 50] single-feature model improved rapidly over the top 50 epochs but eventually stabilized at 60%. This suggests that DAC features have poor ability to distinguish between circRNAs, lncRNAs, and mRNAs. The DNA signatures barely improved in the top 200 epochs, and the F1-weighted signature increased sharply at 200–500 epochs, eventually remaining at 60%.

According to Fig. 2D, dinucleotide-based cross-covariance (DCC) [49, 51] steadily increased with each epoch and finally stabilized at 80%. Dinucleotide-based auto-cross-covariance (DACC) [49, 51] follows the same trend as DCC, but with a larger range of fluctuations and tends to stabilize at 1000 epochs. The composition of k-spaced Nucleotide Pairs (CKSNAP) [49] showed no significant increase in the top 200 epochs, and then experienced a sudden significant rise in the F1-weighted score, stabilizing at 80%, which is generally higher than that of the single-feature model of the DACC. Similarly, the parallel correlation pseudo-dinucleotide composition (PCPseDNC) [52] showed no significant improvement in the first 350 epochs, with a gradual increase in the F1-weighted score that ultimately stabilized at 70%, whereas the pseudo-k-tupler composition (PseKNC) [53, 54] exhibited a similar pattern, with no significant improvement in the first 650 epochs before a steady rise, stabilizing at ~72%.

According to Fig. 2C, in the single-feature model, the 3-mer showed better learning and adaptation abilities during the training process. Specifically, the training curve for the 3-mer feature in the validation set showed a faster convergence rate (stabilized at epoch 50), and the final F-weighted value reached the highest level for all features, close to 85%. Simultaneously, the training curve of the 3-mer features fluctuates less, which further indicates the robustness of the model. Considering that the k-mer feature can capture richer sequence patterns by setting different *k* values, we further compared the feature performances for different *k* values. As shown in Fig. 2E, the performance of the 2-mer was significantly lower than that of the other *k* values, indicating that shorter k-mers cannot effectively capture complex patterns of RNAs sequences. The F1-weighted value of the 3-mer was ahead of the other *k* values and quickly reached a stable state in fewer epochs (after ~50 epochs). The performances of the 4-mer and 5-mer were similar to that of the 3-mer. Therefore, we combined the 3-mer, 4-mer, and 5-mer features for multi-scale fusion to capture complementary information from the different *k*-value features. The F1-weighted value of the model increased to 89.8%.

### Hyperparameter settings

**Figure 3:**
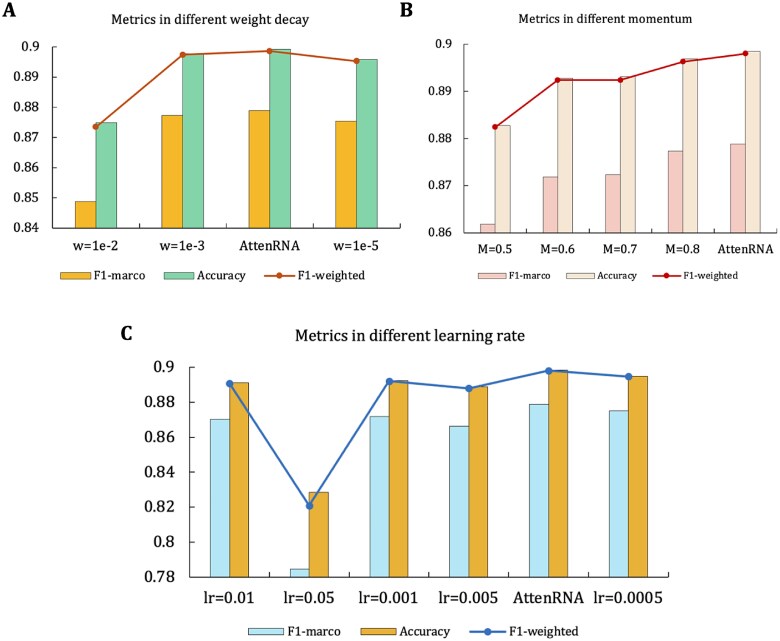
Impact of hyperparameters on model performance. (A) Metrics in different weight decay: comparison of F1-macro, accuracy, and F1-weighted scores under various weight decay settings (*w* = 1e-2, 1e-3, and 1e-5). AttenRNA achieves the highest F1-weighted score with a weight decay of 1e-4. (B) Metrics in different momentum: performance evaluation across different momentum values (*M* = 0.5, 0.6, 0.7, and 0.8). AttenRNA shows optimal performance with the default momentum setting of *M* = 0.9. (C) Metrics in different learning rates: F1-macro, accuracy, and F1-weighted scores are shown for varying learning rates (lr = 0.01, 0.05, 0.001, and 0.0005). AttenRNA demonstrates superior performance at the optimal learning rate of 0.0001.

In this study, we used specific hyperparameters to optimize the performance. AttenRNA was trained using a stochastic gradient descent optimizer with default hyperparameters set to a learning rate (lr) of 0.0001, weight decay (*w*) of 1e-4, and momentum (*M*) of 0.9. These parameters were determined based on experiments to ensure stable training of the model. To explore the influence of the hyperparameters on the model performance, we designed a sensitivity experiment to modify only one hyperparameter each time while keeping the other parameters fixed. The results are shown in Fig. 3:

- Weight attenuation (*w*): We tested three different weight attenuation values of 1e-2, 1e-3, 1e-4, and 1e-5 (see Fig. 3A). The results show that the performance of the model is the best when *w* = 1e-4, and the performance will decrease slightly if the weight attenuation value is further reduced.
- Momentum (*M*): As shown in Fig. 3B, the performance of momentum values 0.5, 0.6, 0.7, and 0.8 was tested against the default value *M* = 0.9. The experimental results showed that when *M* = 0.9, the weighted F1 score of the model reached its highest value.
- Learning rate (lr): As shown in Fig. 3C, we tested learning rates of 0.01, 0.005, 0.0005, and the default value of 0.0001. The experimental results show that the model performance is optimal when the learning rate is 0.0001, whereas a larger learning rate (e.g. lr = 0.01, 0.05, 0.005) significantly reduces the model performance.

These experiments showed that a reasonable setting of hyperparameters, such as the learning rate, weight decay, and momentum, is very important for improving the performance of the model. The final hyperparameter configuration (lr = 0.0001, *w* = 1e-4, *M* = 0.9) made the F1-weighted value of AttenRNA on the validation set 89.8%, which was significantly higher than that of the other configuration models.

### The proposed AttenRNA outperforms baseline models

**Figure 4:**
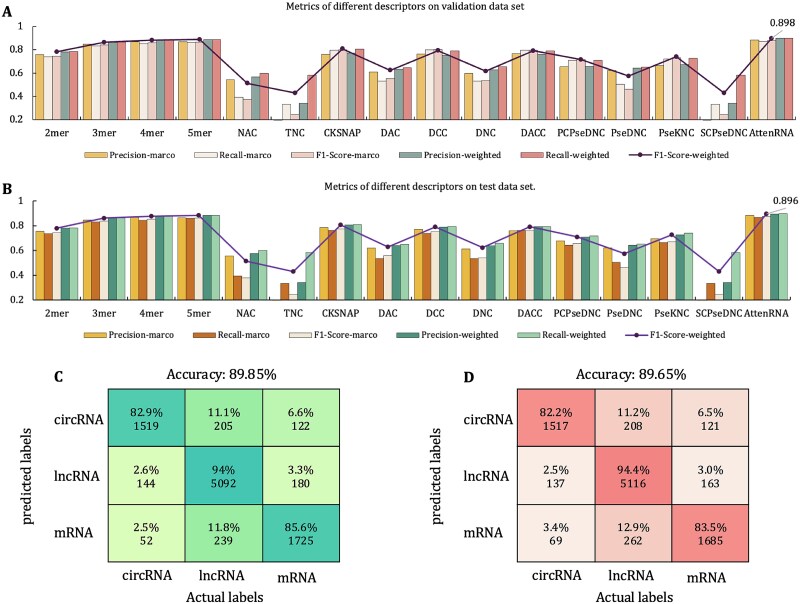
Performance metrics and prediction heatmaps for AttenRNA. (A) Metrics of different descriptors on the validation dataset. The bar chart compares various descriptors using multiple evaluation metrics. The F1-score-weighted is emphasized, showing AttenRNA achieving the highest score of 89.8% among all descriptors. (B) Metrics of different descriptors on the test dataset. Similar to panel A, the bar chart demonstrates the performance of different descriptors on the test data. AttenRNA also achieves the highest F1-score-weighted of 0.896, outperforming all baseline descriptors. (C) Prediction heatmap on the validation dataset. The heatmap visualizes the predicted and actual labels for circRNA, lncRNA, and mRNA. Each cell contains the percentage and count of samples predicted correctly or misclassified. (D) Prediction heatmap on the test dataset. This panel provides a similar analysis to panel C, showing classification results on the test data.

In this study, we comprehensively compared the performance of AttenRNA with that of multiple baseline models on the validation and test sets, and the results are shown in Fig. 4. The performance metrics included precision-macro, Recall-macro, F1-Score-macro, Precision-weighted, recall-weighted, and F1-Score-weighted. AttenRNA significantly outperformed all baseline models in the validation set (Fig. 4A). In particular, AttenRNA achieved 89.8% for F1-score-weighted, which was significantly higher than the highest value of all baseline models (~85%). This suggests that AttenRNA can capture more detailed feature information between RNA types through effective multi-scale k-mers and attention mechanisms. In addition, AttenRNA also showed a high degree of robustness in terms of other indicators (Precision-weighted and Recall-weighted), which remained at ~89%, while other baseline models showed relatively low performance on these indicators, especially traditional features, such as NAC and TNC. Their F1-Score-weighted value are significantly <60%. These results demonstrate the advantages of AttenRNA in the extraction of RNA sequence features. The heat map in Fig. 4C shows the classification performance of AttenRNA in the validation set, with an overall accuracy of 89.85%. In the validation set, the prediction accuracy of circRNAs was the highest (82.9%); however, 11.1% of the circRNAs were misclassified as lncRNAs and 6.6% as mRNA. The accuracy of lncRNA prediction reached 94%; 2.6% of the lncRNAs were incorrectly predicted as circRNAs and 3.3% as mRNA. The accuracy of mRNA prediction was 85.6%; 11.8% of the mRNA were misclassified as lncRNAs and 2.5% as circRNAs.

AttenRNA performed well in the test set, further verifying its generalizability and stability (Fig. 4B). Among all baseline models, the highest F1-Score-weighted score did not exceed 85%, whereas AttenRNA’s F1-Score-weighted score was 89.6%, showing the best performance. In addition, from the perspective of Precision-weighted and Recall-weighted, AttenRNA maintains a high balance, with both indices close to 90%, while baseline models have large fluctuations in these indices, especially the NAC and TNC models. The heat map in Fig. 4D shows the classification performance of the model on the test set, with an overall accuracy of 89.65%. In the test set, the prediction accuracy of the model for circRNAs was 82.2%, among which 11.2% of the circRNAs were misclassified as lncRNAs and 6.5% as mRNA. The prediction accuracy of lncRNAs was 94.4%; only 2.5% of lncRNAs were misclassified as circRNAs, and 3.0% of lncRNAs were misclassified as mRNA. The accuracy of mRNA prediction was 83.5%, and 12.9% of the mRNA were misclassified as lncRNAs and 3.4% as circRNAs. Combining the results of the validation and test sets, AttenRNA performed the best in the RNA classification task, outperforming the baseline model by a significant margin for both the validation and test sets. In particular, the performance in F1-Score-weighted has always been in a leading position, which verifies its powerful prediction ability. The performance of AttenRNA on all indicators showed a high degree of consistency and stability, which further proves the robustness and generalization ability of the model.

### Multi-scale features are more suitable to elucidate the differences between different RNAs

**Figure 5:**
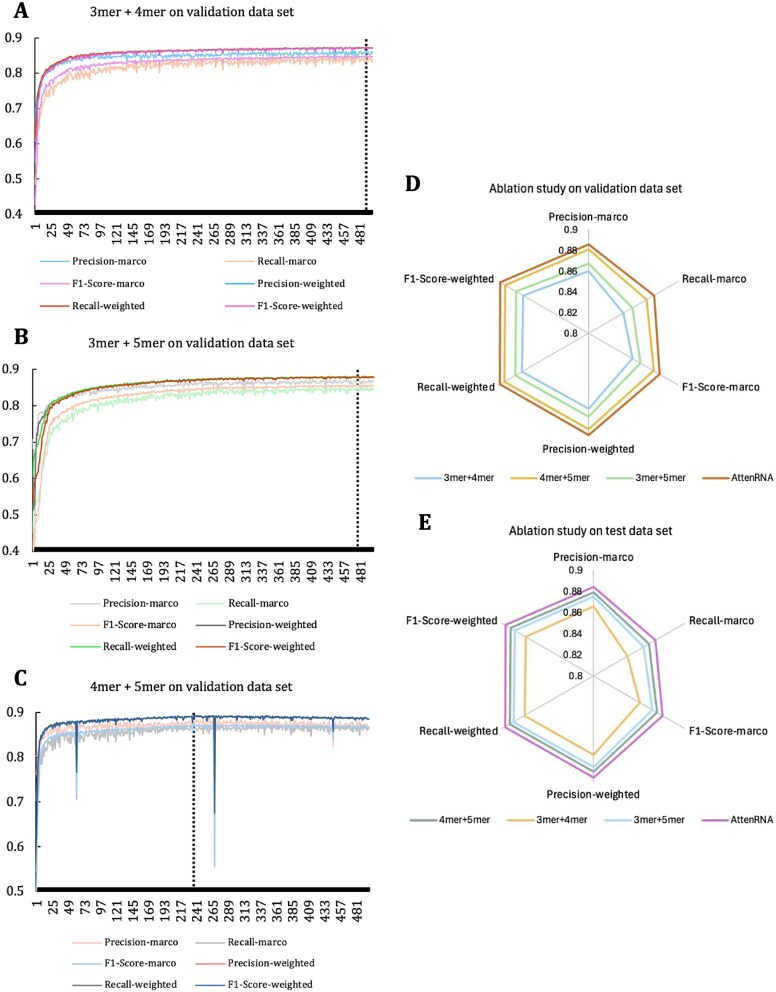
Ablation study. (A) Performance of the 3mer + 4mer combination on the validation dataset. The line chart illustrates various evaluation metrics across 500 epochs. (B) Performance of the 3mer + 5mer combination on the validation dataset. (C) Performance of the 4mer + 5mer combination on the validation dataset. The chart compares the performance of different k-mer combinations (3mer + 4mer, 4mer + 5mer, 3mer + 5mer) and AttenRNA across key metrics such as precision-macro, recall-macro, F1-score-macro, precision-weighted, recall-weighted, and F1-score-weighted. AttenRNA achieves the best overall performance. (D) Radar chart showing the ablation study results on the validation dataset. (E) Radar chart showing the ablation study results on the test dataset. This panel presents a similar analysis as panel D, comparing the performance of different k-mer combinations and AttenRNA. The results consistently indicate that AttenRNA outperforms all k-mer combinations across all metrics.

AttenRNA trains a model using multi-scale k-mers. Figure 2E show the F1-weighted change curve from 2-mer to 5-mer, which cannot adequately capture the intrinsic properties of different RNAs using single-scale models. To solve this problem, we integrated k-mers of different scales as model inputs, including 3-mer + 4-mer (Fig. 5A), 3-mer + 5-mer (Fig. 5B), 4-mer + 5-mer (Fig. 5C), 3-mer + 4-mer + 5-mer (AttenRNA), and compared their performances (Fig. 5D and 5E). The multi-scale k-mer improves the model performance compared to the single-scale k-mer. Specifically, AttenRNA achieved an F1 weighted score of 89.8% in the validation set. This was an improvement of 3.39%, 1.63%, and 1% over using single 3-mer, 4-mer, and 5-mer features, respectively. It also showed improvements of 2.53%, 0.55%, and 1.76% over the 3-mer + 4-mer, 4-mer + 5-mer, and 3-mer + 5-mer combinations, respectively. These results suggest that information from different k-mer scales is complementary, allowing the model to learn more informative feature representations. Shorter k-mers (3-mer) tend to capture local sequence patterns, while longer k-mers (5-mer) are better suited for modelling long-range contextual dependencies. Each k-mer scale contributes uniquely to characterizing RNA sequences. By integrating 3-mer, 4-mer, and 5-mer features, the model leverages multiple sources of information, enabling the deep learning framework to capture their underlying associations and interactions more effectively. As a result, the model achieves improved ability to distinguish between different RNA types.

**Figure 6:**
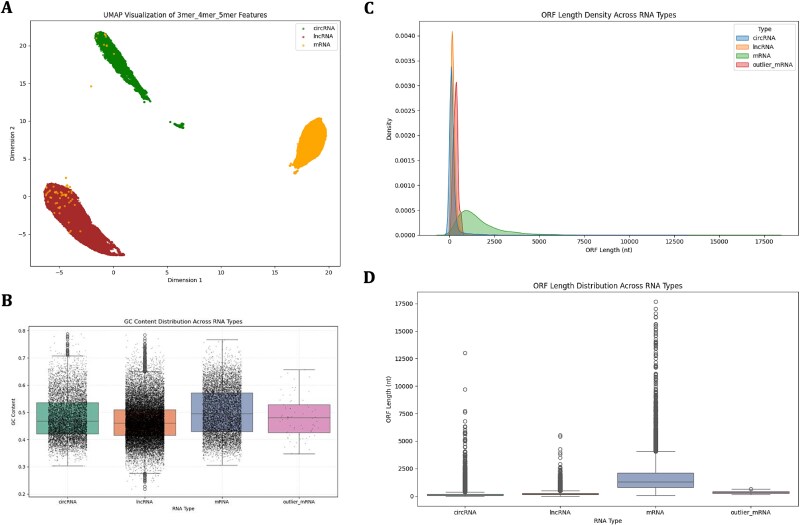
UMAP visualization of multi-scale k-mer features reveals clustering of RNA types, with supplementary sequence analysis of atypical mRNAs. (A) UMAP visualization showing 2D clustering of multi-scale k-mer features for circRNA, lncRNA, and mRNA. (B) GC content distribution of circRNAs, lncRNAs, mRNAs, and outlier mRNAs. Mean GC content: circRNA = 0.481, lncRNA = 0.466, mRNA = 0.502, outlier mRNA = 0.482. (C) Kernel density estimate of ORF lengths distributions across circRNAs, lncRNAs, mRNAs, and outlier mRNAs. (D) Box plot showing ORF length statistics across circRNAs, lncRNAs, mRNAs, and outlier mRNAs.

To further verify the effectiveness of the multi-scale k-mer, we performed a dimensionality reduction visualization analysis on the multi-scale k-mer features using UMAP technology. UMAP is a nonlinear dimensionality reduction method that can preserve the local and global structures of high-dimensional data and is suitable for the visualization of large-scale feature spaces. The experimental results showed that the three human RNA types (Fig. 6A, circRNA—green, mRNA—brown, and lncRNA—orange) formed distinct clusters in two-dimensional space after dimensionality reduction. Specifically, the three RNA types showed a clear separation trend in the projection space, indicating that the multi-scale k-mer could capture specific information of each RNA type. This phenomenon further supports the effectiveness and advantages of multi-scale k-mers for distinguishing between different RNA types. However, we observed that a small subset of mRNA samples appeared in the circRNA and lncRNA clusters (Fig. 6A). To further investigate the outlier mRNA samples observed in the UMAP embedding space, we extracted those mRNAs that appeared clustered with circRNAs and lncRNAs (Fig. 6A). We first examined three RNAs GC content and found that the average GC ratio of outline mRNAs was significantly lower than that of typical mRNAs, and more closely resembled that of circRNAs and lncRNAs (Fig. 6B). Furthermore, we calculated the longest open reading frame (ORF) length for each sequence. Outlier mRNAs displayed substantially shorter ORFs than normal mRNAs (Fig. 6C), with distributions highly similar to circRNAs and lncRNAs (Fig. 6D). These findings suggest that these outline mRNAs exhibit atypical sequence and structural features, including lower GC content and shorter ORF lengths, which are more similar to circRNAs and lncRNAs. The results highlight the value of sequence-based representation in distinguishing RNA subtypes.

### Adaptability of AttenRNA across species

To further assess the effectiveness of the proposed multi-scale k-mer and attention mechanism framework (AttenRNA) in classifying different RNA types, we applied AttenRNA to mouse RNA data. All mouse RNA samples were processed using the same pipeline to ensure consistency in feature representation. AttenRNA demonstrated stable performance on the mouse dataset, achieving a weighted F1-score of 83.89% on the validation set (at epoch 1238) and 83.38% on the test set. Compared to the human dataset, where the model achieved 89.8% and 89.6% weighted F1-scores, the slight decrease in performance may be attributed to species-specific sequence variations. However, the relatively high classification accuracy on mouse RNA data suggests that the multi-scale k-mer embedding and attention mechanism can effectively generalize across species. These results further validate the robustness and adaptability of AttenRNA, demonstrating the effectiveness in RNA classification across different species. The consistent classification performance on both human and mouse RNA data highlights its potential for broader transcriptomic applications.

## Discussion and conclusion

In this study, the proposed AttenRNA model demonstrated superior performance in multi-classification tasks involving circRNAs, lncRNAs, and mRNA. Using multi-scale k-mer embedding and attention mechanisms, AttenRNA can efficiently capture short- and medium-range nucleotide patterns in RNA sequences, while dynamically assessing the importance of different features. This method not only improves the classification performance of the model, but also provides a powerful tool for exploring feature differences between different RNA types. The experimental results showed that the F1-weighted AttenRNA in the validation and test sets reached 89.8% and 89.6%, respectively, which were significantly superior to other baseline models, verifying the effectiveness and advancement of the model. Beyond its high performance in human RNA classification, AttenRNA also demonstrated strong cross-species generalization capability. We applied AttenRNA to mouse RNA data, which were obtained from the same database and processed using the same pipeline to ensure consistency. The AttenRNA-mouse achieved a weighted F1-score of 83.89% on the validation set (at epoch 1238) and 83.38% on the test set, confirming the robustness in recognizing circRNAs, lncRNAs, and mRNAs across species. These results confirm that the AttenRNA framework remains effective across different species, demonstrating its adaptability to RNA classification tasks in diverse biological contexts.

The strength of AttenRNA lies in its designed multi-scale k-mer strategy and the application of attention mechanisms. The multi-scale k-mer embedding method comprehensively extracted the features of 3-mer, 4-mer, and 5-mer, and mined the potential information of RNA sequences by fusing the features of different scales. This method has significant advantages for distinguishing different RNA types. Simultaneously, the attention mechanism can dynamically focus on important feature regions in RNA, further enhancing the interpretability and robustness of the model. In addition, the high performance of AttenRNA is not only manifested in the multi-classification task, but also shows strong adaptability in analysis, providing potential for further research on the function of RNA and its relationship with disease.

However, despite the excellent performance of AttenRNAs, we recognize some limitations in this study. Current models are primarily based on sequence features and lack integration of other crucial information that may affect RNA function, such as secondary structure or epigenetic features, which may limit the comprehensive understanding of RNA function and impact classification accuracy. In addition, there is a class imbalance among circRNA, lncRNA, and mRNA in the training set, which may affect the model’s classification performance and generalization ability. In the future, we will attempt to integrate multimodal data to further improve the classification performance and applicability of this model. Moreover, we will further optimize the efficiency of the model to better adapt to the analytical requirements of large-scale RNA datasets. At the same time, we plan to extend AttenRNA to other RNA classification tasks to provide a more powerful tool for the comprehensive analysis of the function and biological significance of RNA. Finally, we plan to collaborate with wet-laboratory studies to evaluate the biological significance of the predicted classifications and enhance the practical utility of the model in RNA research. In conclusion, the AttenRNA model demonstrated significant advantages in RNA classification, providing a new perspective for RNA classification research. At the same time, we also recognize the limitations of the model and put forward a feasible improvement plan research direction. This work will help promote the further development of RNA analysis.

**Key Points**

- AttenRNA is a multi-class classification framework designed to distinguish mRNAs, lncRNAs, and circRNAs by integrating multi-scale k-mer embeddings and attention mechanisms.
- The model achieved high weighted F1 scores (89.8% validation, 89.6% test), demonstrating excellent performance and robustness.
- AttenRNA generalizes well to cross-species datasets, achieving F1 scores >83% on mouse RNA data.
- This work provides a scalable and accurate tool for systematic RNA classification.

## Data Availability

The data, code, and models from this study are openly accessible on GitHub (https://github.com/lijingtju/AttenRNA). This repository enables researchers to access the datasets, utilize the code, invoke the models developed, and conduct predictions.
